# Early initiation of electrical stimulation paired with range of motion after a volumetric muscle loss injury does not benefit muscle function

**DOI:** 10.1113/EP090630

**Published:** 2022-09-30

**Authors:** Alec M. Basten, Christiana J. Raymond‐Pope, Daniel B. Hoffman, Jarrod A. Call, Sarah M. Greising

**Affiliations:** ^1^ School of Kinesiology University of Minnesota Minneapolis Minnesota USA; ^2^ Department of Physiology and Pharmacology University of Georgia Athens Georgia USA; ^3^ Regenerative Bioscience Center University of Georgia Athens Georgia USA

**Keywords:** muscle function, neuromusculoskeletal injury, range of motion, skeletal muscle

## Abstract

Volumetric muscle loss (VML) injury occurs when a substantial volume of muscle is lost by surgical removal or trauma, resulting in an irrecoverable deficit in muscle function. Recently, it was suggested that VML impacts whole‐body and muscle‐specific metabolism, which might contribute to the inability of the muscle to respond to treatments such as physical rehabilitation. The aim of this work was to understand the complex relationship between physical activity and the response to rehabilitation after VML in an animal model, evaluating the rehabilitation response by measurement of muscle function and whole‐body metabolism. Adult male mice (*n* = 24) underwent a multi‐muscle, full‐thickness VML injury to the gastrocnemius, soleus and plantaris muscles and were randomized into one of three groups: (1) untreated; (2) rehabilitation (i.e., combined electrical stimulation and range of motion, twice per week, beginning 72 h post‐injury, for ∼8 weeks); or (3) rehabilitation and restriction of physical activity. There was a lack of positive adaption associated with electrical stimulation and range of motion intervention alone; however, maximal isometric torque of the posterior muscle group was greater in mice receiving treatment with activity restriction (*P* = 0.008). Physical activity and whole‐body metabolism were measured ∼6 weeks post‐injury; metabolic rate decreased (*P* = 0.001) and respiratory exchange ratio increased (*P* = 0.022) with activity restriction. Therefore, restricting physical activity might enhance an intervention delivered to the injured muscle group but impair whole‐body metabolism. It is possible that restricting activity is important initially post‐injury to protect the muscle from excess demand. A gradual increase in activity throughout the course of treatment might optimize muscle function and whole‐body metabolism.

## Abstract (highlights)

- **What is the central question of this study?**
  First, how does physical rehabilitation influence recovery from traumatic muscle injury? Second, how does physical activity impact the rehabilitation response for skeletal muscle function and whole‐body metabolism?
- **What is the main finding and its importance?**
  The most salient findings were that rehabilitation impaired muscle function and range of motion, while restricting activity mitigated some negative effects but also impacted whole‐body metabolism. These data suggest that first, work must continue to explore treatment parameters, including modality, time, type, duration and intensity, to find the best rehabilitation approaches for volumetric muscle loss injuries; and second, restricting activity acutely might enhance rehabilitation response, but whole‐body co‐morbidities should continue to be considered.

## 1 INTRODUCTION

Physical rehabilitation (i.e., the intentional prescription of general and/or task‐specific regular physical activity with the goal of improving function) is an effective process that uses movement and planned exercise to correct a range of musculoskeletal injuries and disorders (Greising et al., 2020). For many musculoskeletal injuries, specific evidence‐based guidelines are available for prescription of physical rehabilitation (Jarvinen et al., 2007). Guidelines are based on the robust ability of skeletal muscle to adapt to mechanical and chemical cues. For example, after a hamstring injury (e.g., strain) the rehabilitation guidelines are broken down into three phases of healing, with clear recommendations and benchmarks for progression to subsequent phases (Heiderscheit et al., 2010). Briefly, rehabilitation after hamstring injury results in adaptations to the local muscle (i.e., hypertrophy) and the whole body, such as improvements in cardiovascular or metabolic function. Unfortunately, some injuries do not currently have established physical rehabilitation guidelines. One such example is volumetric muscle loss (VML) injury, which has no standard of care to address the skeletal muscle specifically, either surgically or with physical rehabilitation (Greising et al., 2020; Greising et al., 2016). It is well accepted that VML injury is an irrecoverable loss of muscle attributable to surgery or trauma (Garg et al., 2015; Grogan et al., 2011) and is characterized by chronic inflammation, pathological fibrosis and, most distinctively, a permanent loss of force‐producing capacity (Corona et al., 2016; Greising et al., 2017; Hurtgen et al., 2016; Nuutila et al., 2017). Recently, it has become apparent that a co‐morbid aspect of chronic VML injuries is the impairment of whole‐body metabolism (Dalske et al., 2021). It is possible that altered metabolism might further impede the capacity of the muscle for functional improvements after injury with or without physical rehabilitation.

Skeletal muscle is paramount to metabolic activity and health, making significant contributions to basal metabolic rate and insulin‐stimulated glucose uptake. During planned physical activity (i.e., resistance and endurance training), energy consumption increases severalfold and improves metabolic function and flexibility over time (Dolezal & Potteiger, 1998). The expectation of rehabilitation is to have a positive influence on the metabolic health of the whole‐body and skeletal muscle (Egan & Zierath, 2013). Although whole‐body and local muscle metabolic changes have been noted after injury, the metabolic adaptability of VML‐injured muscle after physical rehabilitation is still unclear (Greising et al., 2018). In other injuries, such as spinal cord injury, metabolic rate decreases, in part owing to reduced physical activity resulting from functional impairments (Farkas et al., 2021). Reduced metabolic activity predisposes spinal cord‐injured individuals to chronic metabolic co‐morbidities, including obesity and cardiometabolic dysfunction. In the clinic, routine metabolic measurements are not made after various traumatic injuries, including VML, making it difficult to determine effective interventions, not only for the local injured area but also for the whole body.

Clinical reports indicate that VML‐injured patients see only moderate improvements in muscle function before their progression halts, and further physical rehabilitation, no matter the intensity, fails to improve function (Dziki et al., 2016; Garg et al., 2015; Gentile et al., 2014; Mase et al., 2010; Sicari et al., 2014). Likewise, preclinical models using voluntary wheel running (Aurora et al., 2014, 2015; Corona et al., 2013; Hu et al., 2022; Nakayama et al., 2018; Southern et al., 2019; Washington et al., 2021), forced treadmill running (Izadi et al., 2021; Quarta et al., 2017), electrical nerve stimulation, passive range of motion or a combination (Greising et al., 2018) have only shown modest, if any, improvements in contractile adaptation (Greising et al., 2019), with limited understanding about the physiological rationale for the lack of success. It is possible that dysfunction of skeletal muscle and whole‐body metabolism impairs the capacity to respond to various interventions. Experimental models that mimic more accurately the expected reduction in physical activity after traumatic injuries such as VML are needed to provide insight into how the level of activity influences the pathophysiology (Reidy et al., 2021). This is particularly important because, depending on the timing clinically, early treatments, including rehabilitation, are likely to overlap with periods of inactivity; however, to date there are no models to aid this type of investigation.

After some injuries, ranging from acute muscle strain to critical illness, early mobilization has been shown to improve outcomes (Bayer et al., 2017; Miranda Rocha et al., 2017). Most preclinical models using rehabilitation‐like approaches after VML have initiated rehabilitation 7 days post‐injury (Aurora et al., 2014, 2015; Corona et al., 2013; Hu et al., 2022; Nakayama et al., 2018; Washington et al., 2021); however, exact understanding of the initiating time frame has yet to be established fully. To investigate rehabilitation variables better, clarification of how rehabilitation bouts are undertaken is needed. Interventions such as voluntary wheel running rely on unplanned bouts that can vary greatly in timing and duration, although the total number of kilometres could be consistent between animals (Manzanares et al., 2018). Methods that allow for precise oversight of the type, timing, volume and intensity of exercise bouts, such as chronic neuromuscular electrical stimulation, could be key to this understanding (Lowe & Alway, 2002).

This study was designed primarily to evaluate, in a preclinical mouse model, whether an intervention in the form of neuromuscular electrical stimulation and passive range of motion, twice per week for 8 weeks, could improve the recovery of muscle function and whole‐body metabolism over leaving the VML injury non‐repaired. The treatment was started 3 days post‐VML. The secondary objective of this study was to examine how restriction of physical activity affects the efficacy of the selected rehabilitation after VML, determined through muscle function and whole‐body metabolic measurements. To accomplish these objectives, the study design adopted an innovative approach (Reidy et al., 2021) to restrict activity by restriction housing space.

## 2 METHODS

### 2.1 Ethical approval

Adult (∼12 weeks of age) male C57BL/6 mice (*n* = 24) were purchased from Jackson Laboratories (stock #000664; Bar Harbor, ME, USA). Upon arrival, mice were allowed ≥1 week to acclimate before experimentation. Mice were housed under a 12 h–12 h light–dark cycle and provided with ad libitum access to food and water. All protocols and animal care guidelines were approved by the University of Minnesota Institutional Animal Care and Use Committee (protocol #1803‐35671A), in compliance with the Animal Welfare Act and the Implementing Animal Welfare Regulations and in accordance with the principles of the *Guide for the Care and Use of Laboratory Animals*.

### 2.2 Study design

All mice received an unilateral multi‐muscle, full‐thickness VML injury to the gastrocnemius, soleus and plantaris muscles, as previously described (Dalske et al., 2021; Greising et al., 2018; McFalin‐Figueroa, 2022; Southern et al., 2019). Mice were randomized into three experimental groups: VML; VML ROM‐Estim; and VML ROM‐Estim Restricted. Mice randomized to receive rehabilitation underwent combined range of motion and electrical stimulation (ROM‐Estim) twice per week for 8 weeks. Mice with restricted activity (Restricted) were housed in confined cages shown to limit ambulatory activity by ∼50%. The ankle joint range of motion was evaluated at the terminal time point, 8 weeks post‐VML. At ∼6 weeks post‐VML, all mice underwent evaluation of 24 h whole‐body metabolism and physical activity, as previously described (Dalske et al., 2021). At the terminal time point, 8 weeks post‐VML, contractility of the posterior muscle compartment was evaluated, and the muscles of the hindlimb were harvested before the mice were killed by pentobarbitone overdose (>100 mg/kg, s.c.). Skeletal muscles were snap‐frozen in liquid nitrogen or frozen in 2‐methylbutane cooled by liquid nitrogen and stored at −80°C for future analysis.

### 2.3 Volumetric muscle loss surgery

A multi‐muscle, full‐thickness VML injury to the soleus, plantaris and gastrocnemius muscles was surgically created unilaterally to the left leg (Dalske et al., 2021; Greising et al., 2018; Southern et al., 2019). Mice were administered buprenorphine sustained release (2.0 mg/kg, s.c.) ∼2 h before surgery. Briefly, mice were anaesthetized (isoflurane inhalation 1.5–2.0%; Piramal Critical Care, Bethlehem, PA, USA), and a posterior incision was made to expose the hindlimb muscles. The posterior muscle compartment of the left leg was isolated, and a small metal plate was inserted between the tibia and the soleus muscle. A 4 mm punch biopsy was used to excise ∼15% of the muscle volume (19.5 ± 4.5 mg) from the middle third of the muscle compartment. The skin was closed, and the mice were allowed to recover fully. Immediately after the VML surgery, the mice were randomized into experimental groups.

### 2.4 Passive range of motion and electrical stimulation protocols

Two groups of mice underwent combined passive range of motion and electrical stimulation for the duration of the study. The protocols were based on previous work (Greising et al., 2018). The first bout occurred 72 h after VML surgery, and all mice underwent two bouts per week, each lasting 30 min, for 8 weeks. The final bout was 48 h before the terminal evaluations of muscle contractility. Briefly, at each bout, the mice were anaesthetized (isoflurane inhalation 1.5–2.0%) and placed on a custom‐built platform on their right side. Body temperature was maintained at 37°C for the duration. The knee and hip were stabilized mechanically at 90°, and the left foot was initially placed at a right angle on the footplate, in a neutral position. The footplate was attached to a force transducer of the dual‐mode muscle lever system (300C‐LR; Aurora Scientific, Aurora, ON, Canada). Platinum–iridium needle electrodes were placed subdermally on either side of the sciatic nerve. Optimal placement and current (∼1.4 mA, range 0.8–2.6 mA) were confirmed by a series of 1 and 40 Hz stimulations. Fully computer controlled, the ankle joint was moved passively through 40° of motion in the dorsi‐ and plantar‐flexion direction, with 20° of motion in each direction, then stimulation (0.5 ms pulse width) was delivered with the ankle joint at a neutral position, and the series was repeated over 10 s intervals, for 30 min. For the first 4 weeks of rehabilitation, stimulation was delivered at 30 Hz, 50% duty cycle; this was increased to 45 Hz, 25% duty cycle for the final 4 weeks.

### 2.5 Restricted‐activity housing

Mice were assigned to restricted (*n* = 8) or standard (*n* = 16) cages immediately after recovery from VML surgery and remained for the duration of the study. Dimensions for the small and standard cages were 12 cm × 8.5 cm × 6.3 cm (singly housed) and 28 cm × 18 cm × 12.5 cm (three mice per cage), respectively. All mice in restricted cages received rehabilitation.

### 2.6 Whole‐body physical activity and metabolic assessment

Metabolic and physical activity data were collected using the comprehensive lab animal monitoring system (CLAMS; Columbus Instruments; Columbus, OH, USA) and data examination tool (Clax, v.2.2.15; Columbus Instruments), as previously described (Dalske et al., 2021). All mice were placed in CLAMS at ∼6 weeks post‐VML injury for 48 h consisting of a period of acclimation (the first 24 h) and data collection (the final 24 h). Data were analysed using a customized code created in MATLAB to quantify average data for 24 h, 12 h active and inactive periods, and a moving average over 24 h for metabolic rate and respiratory exchange ratio (RER). Lipid and carbohydrate oxidation rates (in grams per minute) were calculated using the following equations: lipid oxidation = (VO_2_ × 1.695) − (VCO_2_ × 1.701); and carbohydrate oxidation = (VCO_2_ × 4.585) − (VO_2_ × 3.226); VO_2_ volume of oxygen, VCO_2_ volume of carbon dioxide.

### 2.7 Range of motion and in vivo muscle function

**FIGURE 1:**
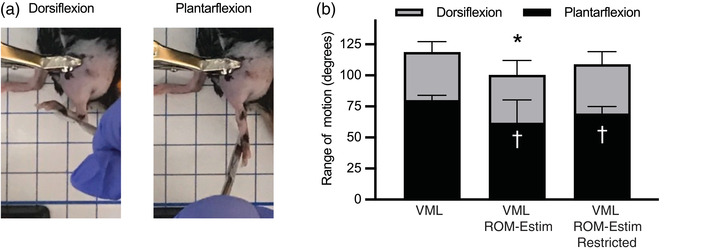
Evaluation of range of motion about the ankle joint. (a) Representative digital images of plantar‐ and dorsi‐flexion. To determine the range of motion about the ankle, visible markers were placed on the lateral tibial plateau, lateral malleolus and distal metatarsal. Quantification of joint angles was determined at extremes of plantar‐ and dorsi‐flexion. (b) The total range of motion was most impaired in the group undergoing rehabilitation alone, compared with VML alone (*P* = 0.001). Additionally, the plantarflexion range of motion was significantly impaired in both rehabilitation groups (*P* ≤ 0.0001). There was no difference in dorsiflexion (*P* = 0.934). *Significantly different from total range of motion in VML alone group. †Significantly different from plantarflexion range of motion in VML alone group. Abbreviations: Restricted, restricted housing; ROM‐Estim, range of motion and electrical stimulation; VML, volumetric muscle loss

Measurement of the range of motion of the involved limb was collected terminally for all mice. Evaluation of ankle joint range of motion was modified from previous work (Garlich et al., 2010). Briefly, mice were anaesthetized (isoflurane 1.5–2.0%), and body temperature was maintained at 37°C for the duration of the procedure. The hair was removed from the hindlimb, and the mouse was positioned on its side against a flat backboard, with the tail secured in line with the spine using tape. The knee joint was clamped into a fixed position. The hip joint of the injured hindlimb was positioned at ∼90° of flexion. Visible markers were placed on the lateral tibial plateau, lateral malleolus and distal metatarsal (Figure 1a). A digital camera was positioned horizontally, directly above the mouse. The foot was moved manually from the resting position to dorsiflexion until a noticeable increase in resistance to ankle motion was detected and held in that position while a digital image was acquired. This process was then repeated for plantarflexion. Exact angles for the range of motion were analysed for all images using FIJI (Schneider et al., 2012).

Evaluation of isometric torque was conducted terminally, 8 weeks post‐VML injury, immediately following evaluation of range of motion. While mice were still anaesthetized (isoflurane inhalation 1.5–2.0%), and body temperature was maintained at 37°C for the duration of the muscle function procedure. The mouse foot was stabilized at 90° (neutral position) to a footplate attached to the servomotor, and two platinum–iridium needle electrodes were placed beneath the skin on either side of the sciatic nerve. To measure passive torque, the servomotor passively rotated the foot from the neutral position to 20° plantarflexion, back to neutral, then to 20° dorsiflexion under computer control, and the force resisting movement was quantified. To measure active torque, the peroneal nerve was severed to eliminate activation of the anterior compartment. Then a current was applied, which increased incrementally by 0.2 mA until peak isometric torque. The force–frequency relationship was measured as a function of stimulation of the muscle at 5, 10, 20, 40, 60, 80, 100, 125, 150 and 200 Hz.

### 2.8 Biochemical analyses

Protein expression of the mitochondrial regulator peroxisome proliferator‐activated receptor gamma coactivator 1‐alpha (PGC1‐α) and collagen content (i.e., hydroxyproline) were examined 8 weeks post‐VML. Upon harvest, the gastrocnemius muscle was portioned into thirds. The distal portion of the gastrocnemius muscle was used to evaluate total collagen content by analysing hydroxyproline, as previously described (Corona et al., 2020; Greising et al., 2018; Hoffman et al., 2021). Briefly, the total hydroxyproline content in the sample was divided by 0.125, representing the proportion of collagen that is hydroxyproline. All samples were run in duplicate and averaged. The proximal third was used for protein expression. Briefly, using a glass pestle tissue grinder, the muscle was homogenized in 10 mM phosphate buffer (pH 7.4) with protease inhibitors (Thermo Fisher, catalogue no. 78440) at a ratio of 1:10 (mg/μl). Homogenate was centrifuged at 10.5*g* for 10 min at 4°C. Total protein content was obtained in triplicate using the Protein A280 setting on a NanoDrop One spectrophotometer (Thermo Fisher) and averaged. Homogenate was then aliquoted and stored at −80°C for immunoblot analyses.

Protein expression of PGC1‐α was determined by immunoblot. Fifty micrograms of protein was separated by 4–15% SDS‐PAGE, transferred onto a low‐fluorescence polyvinylidene difluoride membrane and immunoblotted. Blots were probed for PGC1‐α (Abcam, catalogue no. ab54481, lot no. GR3393001 RRID:AB_881987; 1:1,000) and detected using an appropriate host‐ and isotype‐specific fluorescence‐conjugated secondary antibody, DyLight 800 (Invitrogen, catalogue no. SA5‐10036, lot no. WH3351863). Immunoblots were blocked with 5% bovine serum albumin in Tris‐buffered saline, and primary and secondary antibody dilutions were prepared in 5% bovine serum albumin in Tris‐buffered saline with 0.1% Tween 20. Blots were first imaged using the stain‐free setting on a ChemiDoc MP System (Bio‐Rad Laboratories, Hercules, CA, USA) for total lane protein quantification (Gurtler et al., 2013). Next, blots were imaged using the DyLight 800 channel on the fluorescence setting to visualize the band of interest at the molecular weight noted by the manufacturer's technical information. The intensity of each band was normalized to total protein in each respective lane using Bio‐Rad Laboratories Image Lab software and subsequently used in statistical analyses.

### 2.9 Histological analyses

At the terminal time point, the middle third of the gastrocnemius muscle was saved for histological evaluation. Ten‐micrometre‐thick sections of the mid‐belly of the muscle were cut using a Leica cryostat and microtome. Serial sections were stained using standard procedures for Masson's Trichrome to measure muscle fibre cross‐sectional area, Picrosirius Red (Abcam, catalogue no. ab246832) to quantify fibrosis (i.e., proportion of dense and loosely packed connective tissue), and Oil Red O to evaluate neutral lipid droplets. In all cases, the standardized region of interest was 960 μm × 600 μm (∼1,000 fibres). Images of Oil Red O‐stained slides were obtained using a ×20 objective (0.75 NA, 0.5 μm/pixel resolution) on a TissueScope LE brightfield slide scanner (Huron Digital Pathology, St. Jacobs, ON, Canada). HuronViewer (Huron Digital Pathology) was used subsequently to create and export a single standardized region of interest encompassing the mid‐portion of the muscle with the VML defect area.

Brightfield images of Masson's Trichrome and Picrosirius Red were acquired using a Nikon Eclipse 200 light microscope with at ×10 magnification (E Plan 10×/0.65 OFN20) and a digital camera interfaced to Nikon Elements D software. Picrosirius Red images were acquired with a diascopic cross‐polarized filter using the same objective and magnification.

The cross‐sectional area was quantified for each image using Nikon Elements D software using the Masson's Trichrome‐stained sections. To quantify muscle sections stained with Picrosirius Red solution, images were acquired using polarized light, as previously described (Hoffman et al., 2021); then green, orange/red and yellow thresholds were set in FIJI (Schneider et al., 2012). Briefly, thresholds were set in the L*a*b* color space by adjusting the range of values for all three coordinates. The green threshold was adjusted to 30/255, 0/129 and 0/220, respectively. The red/orange threshold was adjusted to 45/255, 129/255 and 0/220, respectively. The yellow threshold was adjusted to 0/255, 0/129 and 220/255, respectively. Images were then binarized and measured for area. Collagen orientation was quantified using the orange/red/yellow to green ratio, with a higher ratio indicating a greater proportion of densely packed collagen. Data are displayed as areas and collagen packing as a fraction of total collagen area, in accordance with previous work from others (Smith & Barton, 2014). To quantify Oil Red O‐stained muscle sections, similar thresholding methods were used, in this case adjusting the values for L*a*b* coordinates to 0/205, 135/255 and 126/255, respectively.

### 2.10 Statistical analyses

Analysis was performed with JMP Pro statistical software (v.14.2; SAS Institute, Cary, NC, USA). Most dependent variables were analysed by one‐way ANOVA, or two‐way ANOVA for longitudinal range of motion measurements and metabolic rate and RER evaluations across active and inactive periods. When significance was found, Tukey's HSD post‐hoc analysis was used to ascertain significance at specific time points. Simple linear regression was used to evaluate the relationship between ambulation and metabolic rate. Data are presented as the mean ± SD, with individual data points plotted. The statistical significance level was set at *P* ≤ 0.05. During all evaluations, imaging and analyses, the research team was blinded to the experimental groups.

## 3 RESULTS

### 3.1 Animals

Before VML surgery, there was no difference in body mass between groups; all mice were ∼27 g (Table 1) at ∼12 weeks of age. All mice recovered after surgery, and there were no unexpected or adverse events over the 8 week study. At the terminal time point, mice receiving the combined range of motion and electrical stimulation had ∼14% lower body mass than mice that did not (Table 1). Although the mice that received the combined range of motion and electrical stimulation had a lower gastrocnemius mass, there was no difference if normalized to body mass (Table 1).

**TABLE 1:** Muscle and body weights

	**VML**	**VML ROM‐Estim**	**VML ROM‐Estim Restricted**	
**Parameter**	**(*n* = 8)**	**(*n* = 8)**	**(*n* = 8)**	***P*‐value**
Pre‐VML body mass (g)	27.5 ± 1.9	27.3 ± 2.2	26.9 ± 1.6	0.843
Terminal body mass (g)	31.8 ± 2.2	27.2 ± 2.6*	27.3 ± 1.2*	0.002
Injured gastrocnemius mass (mg)	149.6 ± 20.3	117.4 ± 17.6*	111.6 ± 14.9*	0.001
Injured gastrocnemius mass/body mass (mg/g)	4.71 ± 0.68	4.30 ± 0.45	4.10 ± 0.57	0.135
Injured gastrocnemius protein content (%)	9.80 ± 0.97	9.69 ± 0.53	9.43 ± 0.40	0.555
Injured gastrocnemius collagen content (μg/mg)	38.91 ± 15.01	61.39 ± 17.44*	61.83 ± 10.41*	0.007

*Note*. Data are expressed as the mean ± SD.

*Significantly different from VML.

Abbreviations: Restricted, restricted housing; ROM‐Estim, range of motion and electrical stimulation; VML, volumetric muscle loss.

### 3.2 Whole‐body physical activity and metabolic function

**FIGURE 2:**
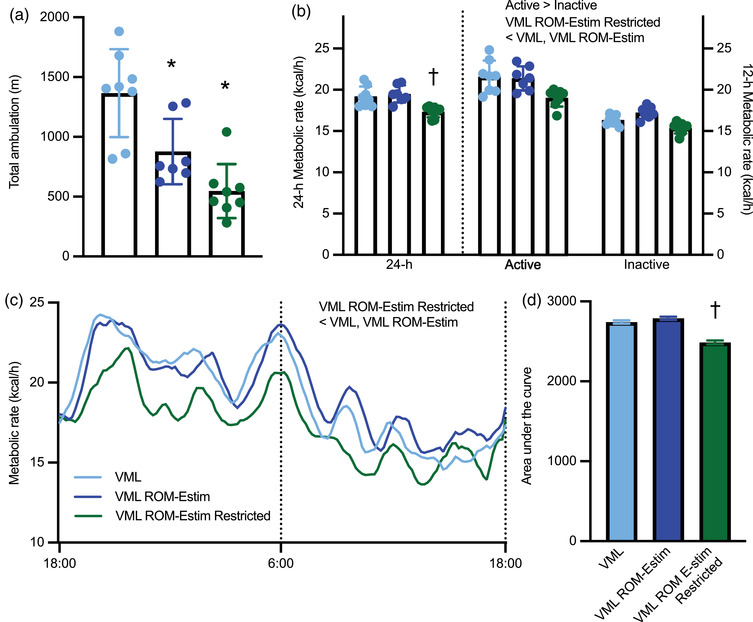
Twenty‐four‐hour metabolic rate and activity 6 weeks after VML. (a) Total ambulation over 24 h, a marker of activity, was reduced in both groups that received rehabilitation (*P* < 0.0001). (b) Over 24 h (*P* = 0.001) and during the 12 h active and inactive periods (main effect of group, *P* < 0.0001), average metabolic rate was reduced only when activity was restricted. As expected, metabolic rate was highest during the active period across all groups (main effect of time, *P* < 0.0001; interaction, *P* = 0.372). (c,d) Quantification of area under the curve also revealed a reduced metabolic rate only for the restricted‐activity group (*P* < 0.0001). *Significantly different from VML alone group. †Significantly different from VML ROM‐Estim group; all significant main effects are noted on the individual graphs. Abbreviations: Restricted, restricted housing; ROM‐Estim, range of motion and electrical stimulation; VML, volumetric muscle loss

At ∼6 weeks after VML, all mice underwent 24 h physical and metabolic activity monitoring. There was a decrease in the 24 h activity of mice in both combined range of motion and electrical stimulation groups (*P* < 0.0001); specifically, ∼36 and ∼60% decline in ambulatory activity with and without restricted housing, respectively, compared with VML injury alone (Figure 2a). The reduced physical activity in restricted housing was reflected in the average metabolic rate (Figure 2b; *P* = 0.001) and quantified as the area under the curve (Figure 2c,d; *P* < 0.0001), indicating decreased whole‐body metabolism in both intervention groups. Ambulation was a predictor of metabolic rate, explaining 20.2% of its variation [*F*(1,21) = 5.332; *P* = 0.031]. As expected, the metabolic rate was greater during the active than the inactive 12 h period (Figure 2b; main effect of time, *P* < 0.0001). Mice that received combined range of motion and electrical stimulation with restricted activity had the lowest metabolic rate over both 12 h periods (Figure 2b; main effect of group, *P* < 0.0001; interaction, *P* = 0.372).

**FIGURE 3:**
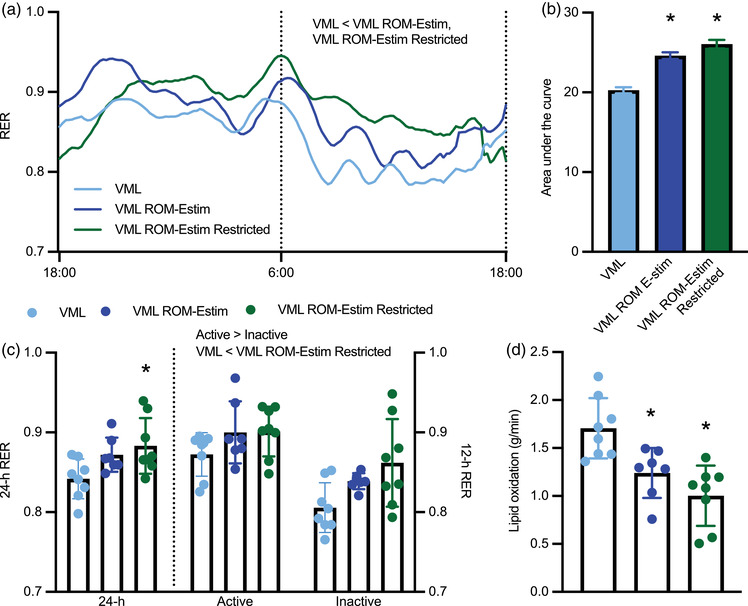
Twenty‐four‐hour RER 6 weeks after VML. (a,b) Independent of activity, both rehabilitation groups had an increase in RER at 6 weeks (*P* = 0.022). (c) All groups had a higher RER during the active period than during the inactive period (main effect of time, *P* ≤ 0.0001), and RER was highest in the active and inactive periods when activity was restricted (main effect of group, *P* = 0.0001; interaction, *P* = 0.818). (d) The increased RER corresponded to reduced lipid oxidation for both rehabilitation groups (*P* = 0.001). *Significantly different from VML alone group; all significant main effects are noted on the individual graphs. Abbreviations: RER, respiratory exchange ratio; Restricted, restricted housing; ROM‐Estim, range of motion and electrical stimulation; VML, volumetric muscle loss

It is expected that a decrease in physical activity will result in a lower RER owing to a reliance on lipids as a fuel substrate. Herein, a higher RER was observed for both combined range of motion and electrical stimulation groups, quantified by the area under the curve (Figure 3a,b; *P* < 0.0001). When averaged, however, RER over 24 h (*P* = 0.022) and during the 12 h active and inactive periods (main effect of group, *P* < 0.0001) was elevated only when physical activity was restricted (Figure 3c). As expected, RER was higher during the active than the inactive period (Figure 3c; main effect of time, *P* < 0.0001; interaction, *P* = 0.818). The increased RER was associated with a decrease in lipid oxidation (Figure 3d; *P* = 0.001). There were no differences in carbohydrate oxidation across groups (*P* = 0.438).

### 3.3 Passive range of motion and passive stiffness about ankle

**FIGURE 4:**
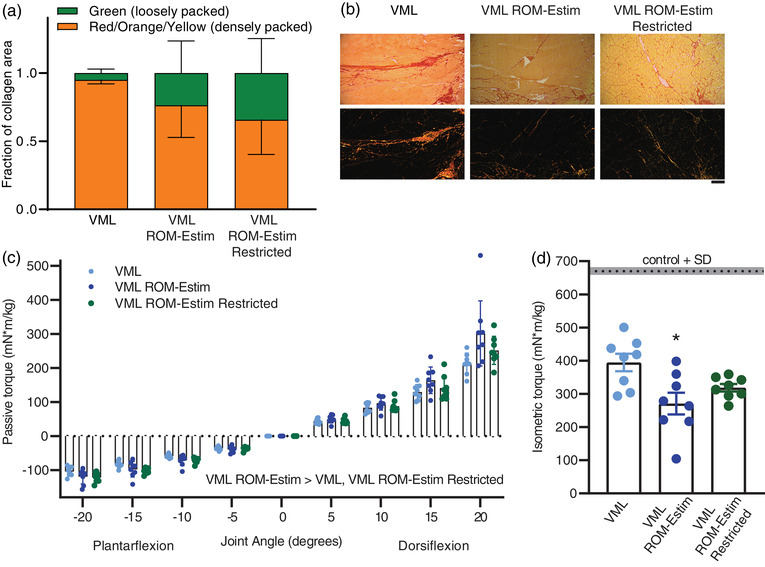
In vivo active and passive function after VML. (a) Loosely packed (green) and densely packed (red/orange/yellow) collagen areas as a fraction of total collagen area within the gastrocnemius muscle were similar across groups (*P* = 0.168). (b) Representative images of the Picrosirius Red staining using a non‐polarized lens (top) or polarized lens (bottom). Polarized images were used to calculate the area of loosely and densely packed collagen. Scale bar: 100 μm. (c) Across 40° of ankle motion, the group that received rehabilitation but was allowed to ambulate freely demonstrated the greatest passive torque (main effect of group, *P* = 0.019; main effect of joint angle, *P* < 0.0001; interaction, *P* < 0.0001). (d) Isometric torque normalized to body mass was impaired in mice that received rehabilitation and were able to ambulate freely, whereas mice that underwent rehabilitation with restricted activity demonstrated a similar torque to the VML alone group (*P* = 0.008). For comparison, data from previously published age‐ and sex‐matched control mice (Dalske et al., 2021) are indicated by the grey band to highlight the VML‐induced impact on function. *Significantly different from VML alone group; all significant main effects are noted on the individual graphs. Abbreviations: Restricted, restricted housing; ROM‐Estim, range of motion and electrical stimulation; VML, volumetric muscle loss

The total range of motion about the ankle was significantly lower in mice receiving treatment without restricted activity compared with VML alone (Figure 1b; *P* = 0.001). This difference can be accounted for by decreased ankle plantarflexion (*P* < 0.0001), because dorsiflexion was not different between groups (*P* = 0.934). Total collagen content within the muscle remaining after injury was greatest in both groups receiving combined range of motion and electrical stimulation (Table 1; *P* = 0.007). The area of loosely packed and densely packed collagen was measured histologically (Figure 4a,b). The total area of loosely packed collagen was not different between groups (*P* = 0.451). Interestingly, the combined range of motion and electrical stimulation groups had less densely packed collagen area than those that did not receive treatment (*P* = 0.011), and restriction of activity resulted in less total (i.e., sum of dense and loosely packed) area of collagen than no treatment (*P* = 0.043). The ratio of densely to loosely packed collagen was ∼70% greater in mice that did not receive range of motion and electrical stimulation (*P* = 0.011), possibly suggesting that a denser fibrous scar forms when there is no intervention. The collagen packing was also evaluated as a fraction of total collagen area, and although not significantly different (Figure 4a; *P* = 0.168), both groups that received treatment had a higher fraction of loosely packed collagen.

The torque developed while passively resisting ankle range of motion was also calculated in 5° ranges, spanning 20° of plantar‐ and dorsi‐flexion. Passive in vivo torque about the ankle joint increased in parallel to total collagen and range of motion (Figure 4c), with the most impact on stiffness in the range of motion and electrical stimulation alone group (main effect of group, *P* = 0.019). There is a known difference across the span of joint angles (main effect, *P* < 0.0001). Only at 20° of dorsiflexion were individual differences across groups noted, with the range of motion and electrical stimulation alone group having the greatest passive stiffness (interaction, *P* < 0.0001). Thus, restriction of physical activity was protective against changes in passive stiffness.

### 3.4 Muscle function and quality

Peak isometric torque of the posterior compartment was assessed terminally (Figure 4d). The range of motion and electrical stimulation alone impaired torque after VML, but restriction of activity mitigated some of the torque loss (Figure 4d; *P* = 0.008). Comparing the torque deficit with the VML alone group, the range of motion and electrical stimulation alone group had a deficit of 36%, and the addition of activity restriction only 19% (*P* = 0.024).

**FIGURE 5:**
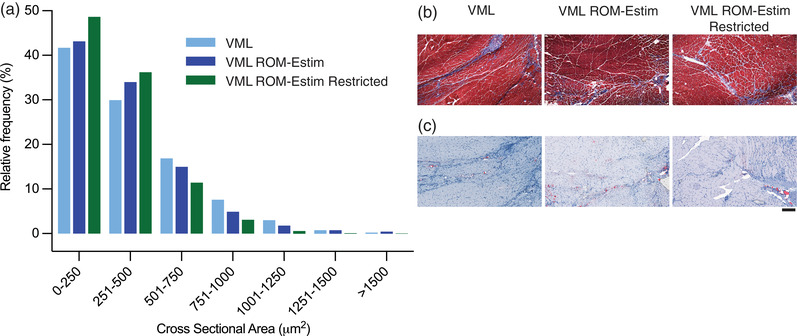
Local muscle metabolic adaptability. (a) Evaluation of the distribution of myofibre cross‐sectional area revealed a higher proportion of smaller fibres in mice receiving rehabilitation that was most evident with activity restriction (*P* < 0.0001). (b) The measurements shown in panel (a) were made using Masson's Trichrome staining of the gastrocnemius muscle. (c) The Oil Red‐O staining of the gastrocnemius muscle revealed a similar percentage area of neutral lipid deposition across groups (*P* = 0.849). Scale bar: 100 μm. Significant main effects are noted on the individual graphs. Abbreviations: Restricted, restricted housing; ROM‐Estim, range of motion and electrical stimulation; VML, volumetric muscle loss

Muscle fibre cross‐sectional area was evaluated to support the torque measurements, but mean cross‐sectional area was not different across groups (371.0 ± 102.3 μm^2^; *P* = 0.062). However, evaluation of the distribution of fibre sizes supported a greater proportion of smaller fibres in the range of motion and electrical stimulation groups, which was most pronounced with restricted activity (Figure 5a,b; *P* < 0.0001).

### 3.5 Local muscle metabolic adaptability

The gastrocnemius muscles were also evaluated histologically and biochemically. Supporting limited metabolic adaptability, histological examination indicated that the percentage area of neutral lipid deposition was not different between groups (Figure 5c; *P* = 0.849), with all groups having ∼1.5% area of lipids.

Across groups, there was no difference in protein expression of PGC‐1α, a marker for mitochondrial biogenesis (*P* = 0.938), suggesting that even with the addition of physical activity restriction, metabolic adaptability was compromised after VML.

## 4 DISCUSSION

This work was designed to evaluate range of motion and electrical stimulation as an intervention model for a mouse VML injury. These interventions were selected because they are clinically relevant and translatable for patients with VML injury during early physical rehabilitation care, when mobility and ambulation can be limited. Various preclinical models of physical rehabilitation methods have been implemented after VML, but modest to no improvements are observed in the ability of the muscle to produce force (Greising et al., 2019). Several possibilities could drive the lack of response to the selected rehabilitation herein. First, physiologically, the muscle remaining after injury might not respond adequately to physical rehabilitation stimuli; both local and whole‐body metabolic dysfunction could impact this lack of response. Second, lack of rehabilitation response could be attributable to poor understanding of the timing, volume and intensity of the rehabilitation regimen. Finally, the overall physiological activity level and state of the VML‐injured animals might compound the response to rehabilitation.

The study was also designed to understand the complex relationship between physical activity and the response to physical rehabilitation after VML, evaluating whether restriction of physical activity could optimize goal‐specific rehabilitation (i.e., range of motion and electrical stimulation) responses to improve muscle function. The data show that although restriction of physical activity during rehabilitation with passive range of motion and electrical stimulation was able to attenuate functional impairments, whole‐body metabolism was negatively impacted. This emphasizes that rehabilitation for a VML injury does not happen in isolation and that the entire body and person must also be considered.

In the clinic, the lack of response to rehabilitation protocols for VML‐injured patients has been established (Dziki et al., 2016; Garg et al., 2015). Although attempts to improve muscle function using physical stimulation (i.e., models of rehabilitation) have had little success after VML (Greising et al., 2019), other studies show that the complete absence of physical stimuli can be equally or more detrimental. Combining a regenerative medicine approach (i.e., acellular biological scaffold) with hindlimb unloading, a model commonly used effectively to eliminate physical loading while retaining muscle activation, completely reversed the scaffold‐derived functional improvement (Dziki et al., 2018). This suggests that the absence of all physical loading or stimuli (at the level of the bone) might be detrimental in the context of a VML regenerative medicine strategy. Notably, experimental models such as hindlimb unloading have limited clinical applicability, because they do not fully mimic bedrest or inactivity directly (Reidy et al., 2021). Hindlimb unloading primarily offloads the bone, alters joint mechanics by leaving the posterior compartment shortened, and can induce significant stress in the animal (Morey‐Holton & Globus, 2002). Rodent hindlimb unloading does not adequately model pathologies associated with injury‐ or lifestyle‐induced inactivity, such as reductions in whole‐body physical activity or muscle‐specific activity.

In the present study, we sought to use a less abrasive approach and to control overall physical activity (McKeehen et al., 2013; Novotny et al., 2012). Past work has suggested that VML injury alone is not sufficient to alter the voluntary physical activity of rodents (Dalske et al., 2021). The use of restricted housing herein was able to limit the ambulatory activity after VML injury by ∼60%. Although reduced, rodents were still able to ambulate daily over the 8 week study, ∼0.5 km/day. This limitation in physical activity resulted in a modest rehabilitation‐based improvement in functional recovery. Although the mechanisms driving this response are not entirely clear, it is possible that although the complete absence of loading is detrimental to functional recovery (as in Dziki et al., 2018), overloading might also be detrimental, that is, over loading with electrical stimulation in the present study. Keeping in mind that the cumulative overload from daily activities and rehabilitation interventions all impact the muscle, rehabilitation guidelines and models should be tuned to achieve the balance between stimuli needed to induce cellular responses and rest to ensure that those cellular responses have adequate time to manifest.

Neuromuscular electrical stimulation is known to promote peripheral axon regeneration (English et al., 2014), muscle hypertrophy (Baar & Esser, 1999; Cabric et al., 1987) and fatigue resistance corresponding to increased oxidative enzymatic activity (Sillen et al., 2013; Theriault et al., 1996, 1994) in various injury models. In general, electrical stimulation has also been shown to increase muscle strength (Gondin et al., 2005), but there are some instances in which chronic high‐frequency stimulation can decrease maximal force, increase type I muscle fibre proportions and decrease fibre cross‐sectional area in muscles with intact motor innervation (Kernell et al., 1987). Similar changes are part of the sequela of VML pathology; it is known that injury alone without intervention will result in an increase in the proportion of type I muscle fibres and decrease in fibre cross‐sectional area (Dalske et al., 2021) in parallel with disrupted motor innervation (Corona et al., 2018). Nonetheless, it was not entirely clear how VML‐injured muscle would respond to electrical stimulation. Overall, there was no difference in muscle fibre cross‐sectional area with range of motion and electrical stimulation herein. Electrical stimulation has been used previously in a model of VML to improve a small proportion of functional outcomes up to 4 months (2 months longer than the present study) post‐injury (Greising et al., 2018), and in this context, the use of electrical stimulation was expected to promote hypertrophy and strength of the muscle remaining in a controlled task‐specific approach. Moreover, we considered that a controlled approach might be superior, in terms of exercise prescription, to the use of voluntary wheel running (Aurora et al., 2014, 2015; Corona et al., 2013; Hu et al., 2022; Nakayama et al., 2018; Southern et al., 2019; Washington et al., 2021), which can be completed in several bouts per day that are highly variable across animals. Although the present data did not show a modest functional improvement to range of motion and electrical stimulation alone as previously noted (Greising et al., 2018), there was moderate benefit to the combined activity restriction and rehabilitation.

The modest benefits could be related to the timing of electrical stimulation in the present study. Although timing is one of several variables (i.e., type, volume and intensity) important to physical rehabilitation protocols and models, it is multifaceted. Specifically, it is key to consider factors such as the timing of initiation after the initial traumatic injury, the duration of bout interventions and the number bouts per week. In the present study, electrical stimulation and range of motion began 3 days after the initial VML injury and was conducted in two 30 min sessions per week. Although the early stage after VML is still being elucidated (Dunn et al., 2019), it is understood to be divergent from standard repair signalling cascades of recoverable muscle injuries (Grounds, 2014); to understand the timing for rehabilitation after VML, this is key to appreciate. In the early days post‐VML, the inflammatory signalling at the muscle is heightened and dysregulated (Aguilar et al., 2018; Larouche et al., 2022). The innervation status of the muscle is impacted over this time, with secondary muscle denervation (Sorensen et al., 2021) and chronic motor neuron axotomy (Corona et al., 2018) continuing to increase at ∼21 days post‐VML. Concurrently, early changes in extracellular matrix composition occur, with a shift to a more densely packed fibrotic scar evident by ∼21 days post‐VML (Hoffman et al., 2021), which could support rehabilitation interventions that target only range of motion. After the initial wound stabilization period but before the exacerbation of secondary denervation, more hypertrophic building with electrical stimulation could be beneficial. Overall, a balance is needed in loading and task‐specific rehabilitation, in addition to the specific physiological needs of the muscle after VML. This will require further alterations and understanding of the timing and/or magnitude of rehabilitation after VML injury.

After VML injury, metabolism of the injured muscle and whole body is altered chronically. At the muscle level, there is altered mitochondrial signalling and muscle fibre oxidative capacity, contributing to an overall contractile slowing of the muscle (Chao et al., 2019; Greising et al., 2018). At the whole‐body level, there is impaired metabolic rate and flexibility (i.e., the capacity to transition efficiently between carbohydrates and lipids used as fuel). Altered metabolism after VML is similar to clinical reports of reduced metabolic rate in spinal cord‐injured individuals attributable, in part, to reduced physical activity resulting in decreased lean body mass (Buchholz et al., 2003; Farkas et al., 2019; Monroe et al., 1998). Recent work indicates that the reduced metabolic flexibility post‐VML is driven largely by ∼4% decreased diurnal RER, a measure indicating the fuel substrate used (i.e., carbohydrates and/or lipids) at a given time, that results from increased lipid oxidation (Dalske et al., 2021). The metabolic inflexibility after VML is similar to that of individuals with disease conditions, including type II diabetes and obesity (Goodpaster & Sparks, 2017), in which insulin resistance can contribute to an increased reliance on lipid as fuel. However, it is unclear how whole‐body metabolism could be impacted by rehabilitation after VML. Herein, compared with VML injuries left untreated, diurnal RER increased by ∼3% after rehabilitation and was more pronounced (∼5%) with restriction of physical activity. The increase in RER was driven by a ∼41% reduction in lipid oxidation, suggesting that rehabilitation mitigates the reduction in RER resulting from VML alone, at a cost of decreasing metabolic rate. Although physical activity is not reduced in rodent models of VML, the restricted‐activity model mimics clinical conditions and reflects the metabolic changes reported with other severe injuries. The findings might support restriction of physical activity initially post‐injury, which might protect the muscle by the reduction in loading, while targeting the muscle in a controlled and balanced manner with various treatment protocols.

Endurance‐type exercise and analogous rehabilitation are known to stimulate adaptative responses through both mechanical and molecular cues (e.g., PGC‐1α and AMPK activation). This work confirms previous evaluations that there is a lack of response of PGC‐1α at the muscle after acute electrical stimulation (Southern et al., 2019) and voluntary running (Aurora et al., 2015). Collectively, these findings demonstrate the limited plasticity of the remaining muscle. PGC‐1α has a role in coordinating multiple organ systems for optimal fuel distribution, and a lack of a PGC‐1α signalling response when muscle is in high demand (i.e., after traumatic injury and during exercise) might also cascade into the observed whole‐body metabolic impairments. Specifically, PGC‐1α has an important role in signalling for lipid oxidative adaptations in skeletal muscle, as fatty acid oxidation takes place within the mitochondria. Increasing demand on the system through rehabilitation could further impair the response of PGC‐1α in the muscle. Along with increased inflammatory and fibrotic signalling, this could result in decreased lipid oxidation and increased RER. Ongoing work is needed to understand these complex relationships.

The seemingly contradictory outcomes with respect to muscle mechanical properties and extracellular matrix composition might be explained by the general lack of understanding between the two. Data suggest that in VML‐injured muscle, there is a variable yet significant negative trend between total collagen content and maximal torque (*r* ^2^ = 0.18, *P* = 0.036). Increased collagen content also seems to be associated with increased stiffness, although the trend is non‐significant. Curiously, early initiation of range of motion and electrical stimulation after VML appears to reduce the formation of densely packed collagen, while also increasing total collagen content. Rehabilitation driving an increase in collagen production is consistent with classical papers on early mobilization after muscle injury (Jarvinen & Sorvari, 1975; Lehto et al., 1985), although the reduction of densely packed collagen adds an unexpected complexity. Previous studies have used less severe injury models and often more aggressive rehabilitation approaches than reported here. A plausible explanation could be that passive range of motion inhibits collagen cross‐linking, while the chronically delivered electrical stimulation creates a large inflammatory response resulting in fibroblast accumulation. Thus, it is also possible that the reduction of force and increase in stiffness might not be attributable to collagen content but to fluid pressure in the muscle tissue owing to inflammation. In any case, the use of an anti‐fibrotic agent coupled with rehabilitation might improve force outcomes by simultaneously limiting the extent of fibrosis and encouraging muscle growth and regeneration. Anti‐fibrotic therapy after VML has been successful in significantly reducing collagen accumulation (Corona et al., 2020); however, this has also led to reduced force output, probably owing to a reduction of force transmission. The findings here indicate that physical rehabilitation produces the same outcome with an opposing mechanism of collagen over‐accumulation rather than reduction. Thus, the combinatorial approach (i.e., regenerative rehabilitation) might be able to regulate extracellular matrix remodelling better, while allowing for beneficial muscular adaptations that are typically inhibited by inflammatory and fibrotic processes.

## 5 CONCLUSIONS

Although discouraging, the lack of complete recovery with physical rehabilitation (i.e., range of motion and electrical stimulation) found herein should not be interpreted to mean that all forms of rehabilitation will fail for those impacted by VML, but that the capacity of the muscle and the whole body to adapt is still being explored, and variables such as the type, timing, volume and intensity of commonly used or new rehabilitation techniques require additional study to formalize optimal interventions after VML. Additionally, ongoing work must focus on combined treatment approaches that optimize goal‐specific tasks and improve muscle function.

## AUTHOR CONTRIBUTIONS

Experiments were conducted in the laboratory of Sarah M. Greising. Conception or design of the work: Jarrod A. Call and Sarah M. Greising. Acquisition, analysis or interpretation of data for the work: Alec M. Basten, Christiana J. Raymond‐Pope, Daniel B. Hoffman, Jarrod A. Call and Sarah M. Greising. Drafting of the work or revising it critically for important intellectual content: Alec M. Basten, Jarrod A. Call and Sarah M. Greising. All authors approved the final version of the manuscript and agree to be accountable for all aspects of the work in ensuring that questions related to the accuracy or integrity of any part of the work are appropriately investigated and resolved. All persons designated as authors qualify for authorship, and all those who qualify for authorship are listed.

## CONFLICT OF INTERESTS

None declared.

## Supporting information

Statistical Summary Document

## Data Availability

The datasets used and/or analysed during the present study are primarily presented in the manuscript and are available from the corresponding author on request.
